# Nitrogen stocks and flows in an acid sulfate soil

**DOI:** 10.1007/s10661-020-08697-1

**Published:** 2020-11-06

**Authors:** Markku Yli-Halla, Seija Virtanen, Kristiina Regina, Peter Österholm, Betty Ehnvall, Jaana Uusi-Kämppä

**Affiliations:** 1 Department of Agricultural Sciences, University of Helsinki, P.O. Box 56, 00014 Helsinki, Finland; 2 Drainage Foundation sr., Simonkatu 12 B, 00100 Helsinki, Finland; 3 Natural Resources Institute Finland, Tietotie 4, 31600 Jokioinen, Finland; 4 Åbo Akademi University, Akatemiankatu 1, 20500 Turku, Finland; 5 Present Address: Swedish University of Agricultural Sciences, Skogsmarksgränd 17, 90183 Umeå, Sweden

**Keywords:** Acid sulfate soil, Controlled drainage, Sub-irrigation, N leaching, Nitrous oxide emissions

## Abstract

Besides causing acidification, acid sulfate (AS) soils contain large nitrogen (N) stocks and are a potential source of N loading to waters and nitrous oxide (N_2_O) emissions. We quantified the stocks and flows of N, including crop yields, N leaching, and N_2_O emissions, in a cultivated AS soil in western Finland. We also investigated whether controlled drainage (CD) and sub-irrigation (CDI) to keep the sulfidic horizons inundated can alleviate N losses. Total N stock at 0–100 cm (19.5 Mg ha^−1^) was smaller than at 100–200 cm (26.6 Mg ha^−1^), and the mineral N stock was largest below 170 cm. Annual N leaching (31–91 kg N ha^−1^) plus N in harvested grain (74–122 kg N ha^−1^) was 148% (range 118–189%) of N applied in fertilizers (90–125 kg N ha^−1^) in 2011–2017, suggesting substantial N supply from soil reserves. Annual emissions of N_2_O measured during 2 years were 8–28 kg N ha^−1^. The most probable reasons for high N_2_O emission rates in AS soils are concomitant large mineral N pools with fluctuating redox conditions and low pH in the oxidized subsoil, all favoring formation of N_2_O in nitrification and denitrification. Although the groundwater level was higher in CD and CDI than in conventional drainage, N load and crop offtake did not differ between the drainage methods, but there were differences in emissions. Nitrogen flows to the atmosphere and drainage water were clearly larger than those in non-AS mineral soils indicating that AS soils are potential hotspots of environmental impacts.

## Introduction

At least 10% of Finnish agricultural land is located on acid sulfate (AS) soils (Palko 1994) which are known for soil acidification and associated severe environmental hazards in ecosystems of recipient waters (Dent and Pons 1995). In coastal areas of the Baltic Sea, hazards arise from oxidation of sulfides accumulated in the parent material, which in most cases is sea sediment rich in carbon (C) from cyanobacterial blooms (Bianchi et al. 2000). Abundance of dead organic material in these sediments served as a source of energy for sulfate-reducing bacteria, which converted sulfate in sea water to sulfide that reacted with metal ions to produce metal sulfides such as pyrite (FeS_2_) (Rickard and Luther 2007) or metastable iron sulfides (e.g., Sohlenius and Öborn 2004; Boman et al. 2008). These sulfidic layers are commonly several meters deep and the C and sulfur (S) stocks are thus much more abundant than in other mineral soils (Öborn 1989; Boman et al. 2008). Owing to postglacial land uplift, AS soils of Finland are concentrated to the coast of the Baltic Sea and are accessible for agricultural use. Artificial drainage is a prerequisite for cultivation, but in AS soils it creates horizons with very low pH (< 4) because newly introduced aerobic conditions trigger oxidation of sulfides and production of sulfuric acid in the subsoil horizons that were waterlogged before drainage.

Large stocks of mineral nitrogen (Nmin) have been found in waterlogged AS subsoil (Cg horizon), mostly in the form of ammonium (Paasonen-Kivekäs and Yli-Halla 2005; Šimek et al. 2011), which are associated with slow mineralization of the large organic matter stock. Waterlogged conditions are not favorable for the oxidation of ammonium to nitrate in the Cg horizon, while in the oxidized Bg horizon nitrification is likely to be prevented by severe acidity and aluminum toxicity. Instead of in situ formation, nitrate may also originate from fertilizers or soil organic matter by mineralization and nitrification in the plough layer and subsequent leaching down to the Bg and Cg horizons. It has been postulated that nitrate/nitrite can be chemically reduced to ammonium in the presence of sulfides (Brunet and Garcia-Gil 1996). The fate of the large Nmin stock in AS subsoil is largely unknown, but water quality monitoring in Finland in 1965–2010 (Rekolainen 1989; Tattari et al. 2017) suggested that presence of AS soils in a catchment increases the N load to watercourses.

As sulfur can interfere with C and N cycles, the biogeochemistry of AS soils can be affected, with implications for the greenhouse gas balance. In particular, high nitrous oxide (N_2_O) emissions have been reported from AS soils (Denmead et al. 2010; Macdonald et al. 2011; Petersen et al. 2012). In anoxic conditions, production of N_2_ and N_2_O may be caused by nitrate reduction coupled with oxidation of iron sulfides or ferrous iron (Fe^2+^) (Postma et al. 1991; Schippers and Jørgensen 2002; Vaclavkova et al. 2014; Virtanen 2015). Low pH may further increase the proportion of N_2_O in the end products of denitrification by inhibiting N_2_O reductase (Thomsen et al. 1994).

Acidification of waters caused by AS soils can be partly alleviated by keeping the sulfidic horizon waterlogged and thus preventing oxidation of sulfides. For this purpose, controlled drainage (CD) (e.g., Palko 1994; Österholm et al. 2015), and sub-irrigation associated with controlled drainage (CDI) (Österholm et al. 2015) have been tested in situ in AS fields resulting in at least slightly lower acidity in discharge water (Åström et al. 2007; Johnston et al. 2014; Virtanen et al. 2016). In non-AS fields, controlled drainage can decrease nitrate loading by 18–75% (Ritzema and Stuyt 2015), mainly due to higher evapotranspiration resulting in lower drain discharge but also to more efficient use of nutrients (Wesström et al. 2014; Ritzema and Stuyt 2015). In a field and simulation study in Canada, Jiang et al. (2019) observed that controlled drainage reduced CO_2_ emissions but increased N_2_O emissions due to higher denitrification in a non-acid soil.

Acidification of soil and watercourses caused by cultivation of AS soils has been widely studied but other environmental consequences are less well researched. In order to assess N flows in AS soils, we quantified the N stocks and monitored N uptake by crop yield, leaching of N, and N_2_O emissions in three adjacent fields on an AS soil on the western coast of Finland. We also studied how different subsurface pipe drainage practices affected N loading to watercourses or emissions to the atmosphere. Owing to the large N stock in AS soils, it was hypothesized that (1) N loading and (2) emissions from AS soils are large, and that controlled drainage (CD) and especially sub-irrigation (CDI) lead to (3) less leaching of N and (4) lower N_2_O emissions due to longer anoxic periods in subsoil and lower mineralization of the N stock.

## Materials and methods

### Experimental field and its management

**Fig. 1:**
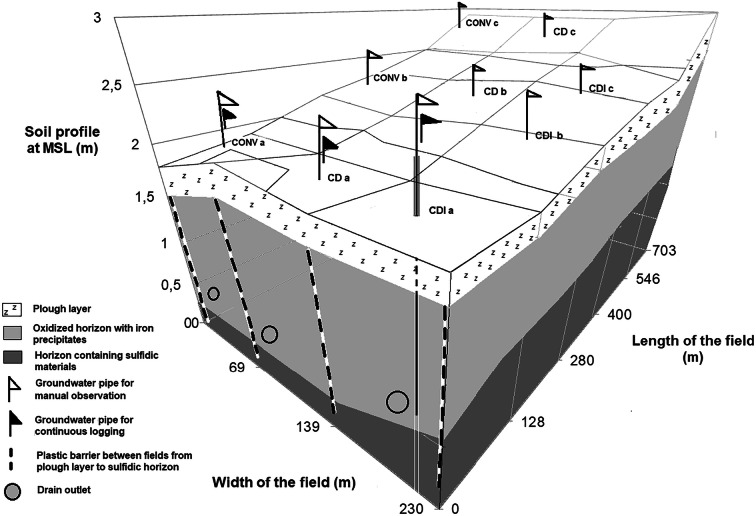
Layout of the experimental area showing the locations of the subsurface drainage pipes, groundwater observation pipes, structures preventing water flow between fields, and soil horizons in the experimental area relative to mean sea level (MSL). The fields have three different water management practices: CONV = conventional subsurface drainage, CD = controlled drainage, CDI = controlled drainage with sub-irrigation

The experiment was established in 2010 on three adjacent fields (total area 18.5 ha) in a polder area of Söderfjärden (63° 0.1896′ N, 21° 35.4747′ E) near Vaasa in Western Finland in 2010. The polder was reclaimed for agriculture in the 1920s and the parent material consists of Holocene sulfide-bearing marine sediments. The area and the experimental setup and treatments (Fig. 1) are described in detail by Österholm et al. (2015). Groundwater (GW) level, concentration of N in drainage water, and grain yield were monitored in 2010–2017. Emissions of N_2_O were measured periodically in 2010–2014 and mechanisms leading to N_2_O emissions were studied in laboratory experiments.

The soil has a silt loam Ap horizon (0–28 cm) while the subsoil consists of silty clay loam. A gleyic color pattern with a strong structure and continuous iron hydroxide coatings is visible throughout the Bg (28–120 cm) and BCg (120–150 cm) horizons. The Cg horizon below 150 cm has a massive structure, with no rust mottles, and contains black sulfidic material inherited from the parent sediment. The soil is classified as Sulfic Cryaquepts (Soil Survey Staff 2014) and Thionic Gleysols (drainic, humic, loamic/siltic) (IUSS Working Group WRB 2015).

The experimental fields were around 80–100 m wide and 710–740 m long. The surface of the lower part of each field, closest to the main drain, is 1.9 m above the mean sea level (MSL) and the edge farthest from the main drain (upper part) is 2.9 m above MSL, the slope being < 0.2% (Fig. 1). The fields had existing subsurface drainage pipes at 1.1 m with control wells (Fig. 1). The following experimental water management systems were practiced in the individual fields: (1) conventional subsurface drainage (CONV), (2) controlled subsurface drainage (CD), and (3) controlled subsurface drainage with additional pumping of water into the control wells during dry periods (sub-irrigation, CDI). In order to have similar drain spacing (26–28 m) in each experimental field, before sowing in May 2010, supplementary drains were installed in the CONV field, where the existing drainage system originating from the 1950s had a wider drain spacing. Although 2010 was meant to be a calibration period by keeping the wells open (i.e., applying CONV) in each field during the growing season, due to drought, additional water was pumped into a closed control well in July by the farmer managing the CDI field. The amount of sub-irrigation in 2011–2014 was 31 mm, 50 mm, 12 mm, and 28 mm, respectively, with 22 mm in 2016. No sub-irrigation was applied in 2015 and 2017 because water was not available, and thus the CD treatment was applied to the CDI field in those years.

In order to prevent bypass flow, all three test fields were hydrologically isolated from each other and from the main drain using vertical plastic sheets from 0.3- down to 1.8-m depth, extending into the impermeable massive parent sediment. Continuous and real-time measuring devices were installed in each field after crop harvest in September 2010. Perforated groundwater (GW) pipes were installed in each field to 2.5-m depth; one for a GW logger (pressure sensor, EHP GWL-600, EHP-Tekniikka Ltd., Oulu, Finland) and three other pipes for manual GW measurements in the lower, middle, and upper part of the field. The manual measurements were made by tape measure. In the lowest section of the fields, the manually and continuously measured groundwater pipes were only about 1 m apart. The mean difference between simultaneous continuous and manual GW measurements was 20 ± 18 cm.

During the growing season, precipitation data were collected with a simple rain gauge near the experimental field by a local farmer or with a RainWise MK III weather station (EHP-Tekniikka Ltd., Oulu, Finland) in the field. Precipitation data from the weather station was not always available due to problems caused by gull excrement. Air temperature was measured with a Vaisala HMP45 sensor in the field. Annual precipitation and air temperature data throughout the year were extracted from the databases at the Finnish Meteorological Institute (Table 1).

**Table 1:** Meteorological data for the Söderfjärden experimental area. All values were extracted from the Finnish Meteorological Institute database except precipitation during the growing season in seven years, which was measured directly in the field. Average precipitation and temperature at Vaasa airport, 10 km from the experimental field, in 1981–2010 was 552 mm and 4.2 °C, respectively

Year	Mean air temperature (3), °C	Annual precipitation (3), mm	Precipitation during growing period (4), mm	Growing degree days (1), °C	Growing season	Length of growing period, days
2010 (5)	–	540	159 (2)	–	8.5.–11.10.	156
2011	5.9	617	304 (2)	1446	16.4.–18.11.	217
2012	4.3	687	199 (1)	1171	23.4.–19.10.	180
2013	5.9	556	260 (2)	1387	2.5.–15.10.	167
2014	6.0	547	193 (1)	1321	17.4.–13.10.	180
2015	6.4	738	399 (2)	1146	19.4.–11.11.	207
2016	5.2	665	455 (2)	1272	28.4.–4.10.	160
2017	5.2	636	173 (1)	1040	16.5.–18.10.	156
Mean	5.7	623	268	1255		181

(1) Measured in the field or (2) next to the field. (3) Measured at Mustasaari, Riimala, 14 km away from the field. (4) Precipitation between sowing and harvesting of the crop. (5) Incomplete experimental year

The area was farmed in a coordinated manner by the three local farmers who own the fields. Spring barley was grown in 2010, 2012, 2014, 2016, and 2017 and spring wheat in 2011, 2013, and 2015. The fields were sown in May, with simultaneous application of NPK mineral fertilizer at the rate of 90 and 110–125 kg N ha^−1^ for barley and wheat, respectively. Crop yield was quantified using an experimental harvester (width 1.5 m), harvesting 15-m-long representative sections of the lower, middle, and upper sections of each field. The grain samples were analyzed for moisture and N concentration. Straw yield was measured in 2010, 2012, and 2013 and analyzed for N in 2010. The straw was incorporated into the soil by ploughing.

### Sampling of soil and water

Soil profiles at the lower and upper section of each field were sampled in autumn 2009 with an auger at vertical depth intervals of 10 cm down to 2.0 m and the pH (1:1) in deionized water was measured in the field (Österholm and Åström 2002). Soil samples were taken at intervals of 20 cm down to 2.0 m from the lower section of each field in May 2012 and analyzed for total C and N by dry combustion (LECO, St. Joseph, MI, USA). Bulk density (BD) of the two uppermost sections, needed for conversion of C and N concentrations to kilograms per hectare, was determined using 0.04-dm^3^ cylinders. Owing to a rather uniform organic C content, it was assumed that deeper layers had the same BD as at 20–40 cm. The plough layer (Ap horizon) and the upper part of the Bg horizon (25–40 cm) in the lower, middle, and upper section of each field were sampled in May 2010–2013 for Nmin. Additionally, the soil was sampled for Nmin analysis at depths of 100–120 cm (lower part of the Bg horizon), 130–150 cm (BCg horizon), and 170–190 cm (Cg horizon) in September 2013. The soil samples were stored frozen until Nmin was extracted with 2 M KCl (Esala 1995). The concentrations of ammonium-N (NH_4_^+^–N) and nitrate-N (NO_3_^−^–N) were determined colorimetrically with an autoanalyzer (Lachat QuickChem AE, Hach, Loveland, CO, USA, or Skalar San++ System, Skalar Analytical B.V., Breda, the Netherlands).

An EHP-UltraSonic Flow monitoring system (EHP-Tekniikka Ltd., Oulu, Finland) was installed in order to monitor the water flow (dm^3^ s^−1^) online, continuously and in real time in the outlet pipe of each field. The system was a combination of an EHP DL-6 data-logger with an amkf12 gsm/gprs-modem, Fluxus 5107 ultrasonic device, 2-m EHP pipe, accumulators, and solar panels. The data logger transmitted the raw data detected by the sensors to an online server (http://www.ehp-data.com). Daily data were manually corrected by removing negative and suspiciously high values, and faulty values measured at low battery current. Upon conversion from cubic decimeter per second to cubic decimeter per second per hectare and millimeter per year, it was assumed that the drainage basin was only the area bordered by the plastic sheet for each field (5.66 ha in CONV, 5.97 ha in CD, and 6.75 ha in CDI).

Representative drainage water samples were collected from November 2010 until December 2017, under thawless moderate to high flow conditions (> 0.1 dm^3^ s^−1^), in spring (April–May; *n* = 16) and autumn (October–December; *n* = 23). These samples were taken from the lowest well of each field. In 2010 and 2012, a total of 7–9 samples per year were taken, while in 2011, and 2013–2017, the annual number was 11–13. The water samples were analyzed for electrical conductivity (EC; SFS-EN 27888:^1994^), Ntot, NH_4_^+^–N, NO_3_^−^–N, and nitrite-N (NO_2_^−^–N). A colorimetric flow injection analysis (FIA) method was used for the determination of NH_4_^+^–N, NO_3_^−^–N, and NO_2_^−^–N according to Finnish standard methods (SFS-EN ISO 11732:^2005^, SFS-EN ISO 13395:^1997^). For determination of Ntot, the water samples were digested with K_2_S_2_O_8_ (ISO 11905-1:^1997^).

The concentration of NO_*x*_^−^–N (sum of NO_3_^−^–N and NO_2_^−^–N) was continuously monitored (Hach-Langen Nitratax sensor) in the lowest control well of CDI from April 2012 until the end of November 2014. In order to discover which soil horizons are the main sources for N leaching, we calculated the daily NO_*x*_^−^–N and GW values and plotted NO_*x*_^−^–N concentration and GW level in the soil profile against the measurement date. From the daily locations of GW in every 20-cm range of a depth in the soil profile, the median of NO_*x*_^−^–N was calculated for spring, summer, and autumn. The other continuously monitored parameters (GW, EC, discharge) were compiled in a similar manner. Negative daily discharge and NO_*x*_^−^–N concentrations < 1 mg L^−1^ were filtered out to exclude the effect of stagnant water in the control well. During floods, the outlets of the drain pipes were below the water surface in the nearby ditch, and ditch water could flow back through the pipes into the field. When the floodwaters receded, water flow from the field through the drain pipes resumed. Surface runoff caused by floodwaters was not measured.

The N load for each field was calculated from grab sampling and continuous monitoring data using the following equation:

$$ \mathrm{Annual}\ \mathrm{N}\ \mathrm{load}\ \left(\mathrm{kg}\ {\mathrm{ha}}^{-1}\right)={C}_w\times Q $$

where *C*_*w*_ is the runoff weighted average N concentration (mg L^−1^) in grab samples taken from the well in the lowest section of each field, and *Q* is the cumulative discharge of measured continuous runoff (L ha^−1^).

### Measurement of N_2_O fluxes and denitrifying enzyme activity

The N_2_O flux was measured with a closed chamber technique in the field experiment during three periods, Oct 2010–Sept 2011, May 2012–May 2013, and June–Aug 2014. Due to limited resources, the measurements were conducted at low frequency and varying intervals; nine times in 2010–2011, 14 times in 2012–2013, and eight times in 2014. Five replicate steel collars were installed in the lower section of each field (Fig. 1), and removed only when necessary for farming operations. A water seal between the edge of the collar and the chamber ensured gas tightness of the chamber (Kanerva et al. 2007). Chamber measurements were carried out using closed aluminum chambers from which four samples were taken during 45 min. Gas samples (20 mL) were taken with BD Plastipak polypropylene syringes (Becton, Dickinson and Company, Franklin Lakes, NJ, USA) and stored in pre-evacuated 12-mL Exetainer glass vials (Labco Ltd., High Wycombe, UK).

The N_2_O concentrations were also measured at depths of 30, 50, and 70 cm in the soil profile on seven occasions in summer 2012. Sampling lines made from 1-mm PTFE tube were installed in the soil at three replicate locations per plot. A sintered polyethylene filter (pore diameter 100 μm) was placed at the sampling depth and a 5-mL sample was drawn through the filter after discarding the first 5 mL.

The gas samples were analyzed for N_2_O with a gas chromatograph (details in Kanerva et al. 2007). A reference gas mixture (AGA Gas AB, Lidingö, Sweden) of known concentrations of N_2_O was used for the calibration curve. The linear response resulting from analysis of the four gas samples taken during the 45-min enclosure period was used for calculating the emission rate. The volume of gas in the chamber was corrected according to the chamber temperature. The annual rates of gas fluxes were calculated with linear interpolation of the daily flux rates between subsequent sampling days for the periods Oct 27, 2010–Oct 26, 2011 and May 28, 2012–May 27, 2013.

The effect of soil pH on denitrifying enzyme activity (DEA) was studied in the Ap (0–20 cm) and Bg (80–100 cm) horizons in CONV. Soil for these analyses was taken in June 2014 as 15 pooled subsamples close to the gas measurement frames of each field. The samples were stored at + 4 °C and sieved (2 mm) just before use. Five replicate 20-g soil samples were weighed into 120-mL incubation flasks and kept in room temperature for an hour. To adjust the desired pH, a 20-mL aliquot of a solution of H_2_SO_4_ or NaOH was added according to a preliminary test. Next, 5 mL of a solution containing glucose (100 mg L^−1^) and KNO_3_ (500 mg L^−1^) were added, and the flasks were closed with butyl rubber septa and evacuated using a vacuum pump. To ensure anaerobic conditions, the evacuation and flushing with helium was performed four times. After the last round of evacuating, the flasks were filled with helium again, the overpressure was released through a needle, and acetylene was added to half the replicates to obtain a final concentration of 10% acetylene in the gas phase. The samples were shaken in a rotary shaker (180 rpm) and 1-mL gas samples were taken at 1 min and 60 min and analyzed as described above.

### Statistical analyses

Parameters monitored at 10- to 30-min intervals were aggregated into daily values by summation (precipitation and discharge) or taking their daily means (GW, EC). Differences in GW between the fields were determined using the Mann-Whitney rank sum test in SigmaPlot 12.3. Differences in Nmin content of the soil at 0–40 cm between the years were tested with one-way ANOVA. Standard deviation (SD) was calculated for the annual grain yields and N offtakes in CONV, CD, and CDI, using the results of the three harvested sections of the respective field as replicates. Standard deviation of N concentrations in water samples was calculated separately for autumn and spring. Statistical analysis of the field greenhouse gas data was performed using the SAS Enterprise Guide software, version 7.1. The effect of drainage treatment on field fluxes of N_2_O was studied using the generalized linear mixed model approach. The estimation technique was restricted maximum likelihood (REML) and the method of estimating degrees of freedom was Kenward-Roger 2. The values of N_2_O were log-transformed to normalize their distribution. Differences in DEA were tested for significance using the independent sample *T* test in IBM SPSS version 22.

## Results

### Carbon and nitrogen stocks and soil pH

**Fig. 2:**
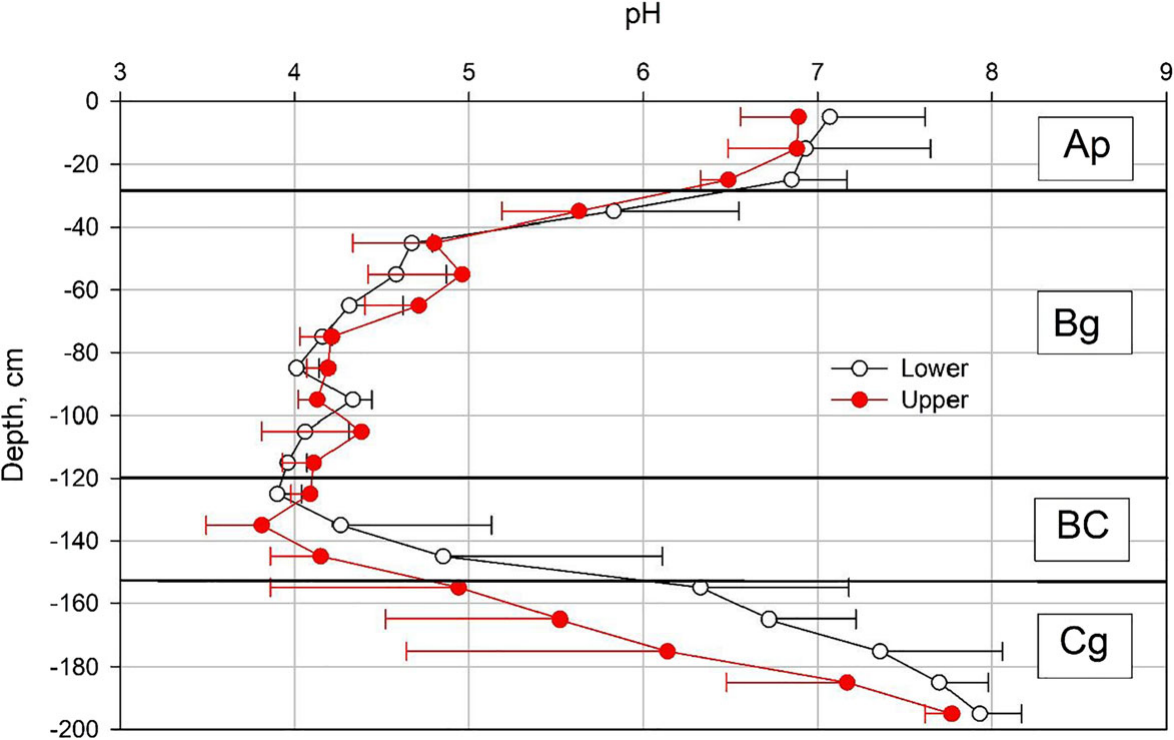
Genetic horizons and soil pH in the upper and lower sections of each field, measured in autumn 2009. The error bars indicate standard deviation calculated for the three fields

**Fig. 3:**
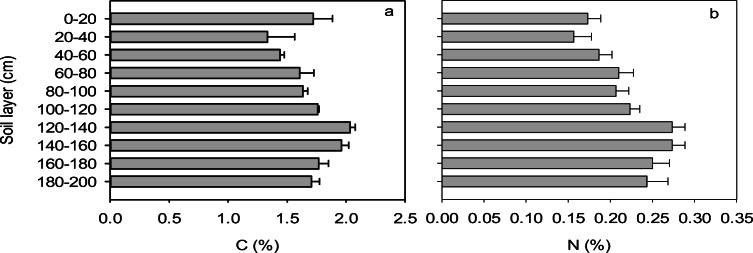
Content of **a** total carbon (C) and **b** total nitrogen (N) in the soil profile (mean and standard deviation of the results of all three fields)

It was found that the natively low pH of the Ap horizon at the site had been elevated over time by heavy liming, whereas the oxidized Bg horizon had a pH between 3.9 and 5.0 (Fig. 2). The pH of the BCg horizon was higher than that in the horizon above, and the pH reached neutrality in the reduced parent sediment (Cg horizon). The concentrations of C and N in the soil profile were rather uniform, being marginally higher in the subsoil at 120–160-cm depth (Fig. 3). Total stocks of C and N were 162 and 19.5 t ha^−1^, respectively, in the uppermost 100-cm layer of soil, and 194 and 26.6 t ha^−1^, respectively, at 100–200-cm depth.

### Mineral nitrogen in soil

There was a decreasing trend in Nmin during the 4-year measurement period. In spring of the last 2 years (2012–2013), Nmin amounted to only about half the value measured earlier (2010–2011) (Table 2). The difference was attributable almost solely to NO_3_^−^–N while the amount of NH_4_^+^–N was nearly constant every spring. There was little difference in Nmin concentrations between the two sampled layers (0–25 cm, 25–40 cm) and therefore their results are not shown separately. The NO_3_^−^–N fraction amounted to 54–78% of the entire Nmin, with the percentage being higher in years with higher amounts of Nmin. There were no significant differences in Nmin at 0–40 cm between the fields (i.e., drainage methods) in any of the 4 years for which Nmin was measured. When the horizons below 40 cm were also analyzed for Nmin in 2013, it emerged that the largest Nmin stock was deeper in the soil than crop roots commonly reach. At 100–120 cm in the oxidized subsoil (Bg), Nmin amounted to 28 kg ha^−1^, with NO_3_^−^–N comprising 86%, while at 130–150 cm (BCg) Nmin was at a similar level (23 kg ha^−1^) but at this depth NH_4_^+^–N started to dominate the pool, comprising 79%. In the reduced subsoil at 170–190 cm (Cg), Nmin increased to 200 kg ha^−1^, all in the form of NH_4_^+^–N.

**Table 2:** Mineral nitrogen (N) content in the 0–40-cm soil layer in May 2010–2013. Each row was tested separately. Means marked with different letters are significantly different (*p* = 0.05)

	2010	2011	2012	2013	*F* value
NO_3_^−^–N, kg ha^−1^	30.3^a^	33.8^a^	15.4^b^	14.5^b^	28.637***, *p* < 0.001
NH_4_^+^–N, kg ha^−1^	12.9^a^	8.4^a^	7.6^a^	10.8^a^	2.401^n.s.^, *p* = 0.09
Nmin, kg ha^−1^	43.2^a^	42.2^a^	23.0^b^	25.3^b^	15.856***, *p* < 0.001

### Water management and groundwater level

**Fig. 4:**
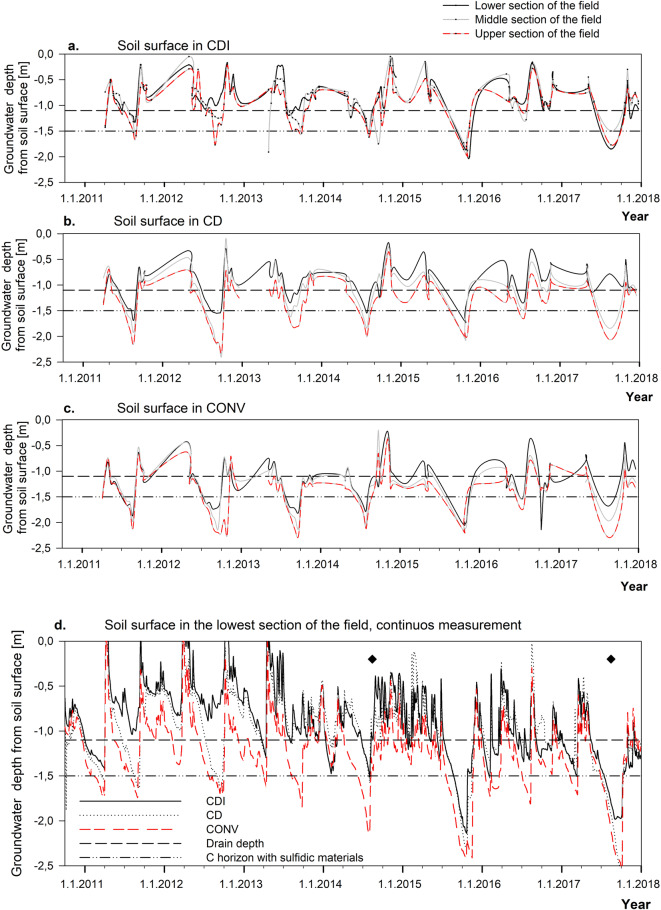
Results of manual groundwater level measurements in the lowest, middle, and upper section of the fields in the **a** sub-irrigated (CDI), **b** controlled drainage (CD), and **c** conventional subsurface pipe drainage (CONV) treatments in 2011–2017. **d** Results of continuous groundwater level measurements in the lowest part of the experimental area in 2011–2017. The diamond symbols indicate date of dredging of the main drain

The GW level varied seasonally rising up to the soil surface after snow melt in spring and due to frequent rainfall events in autumn (Fig. 4). Low GW was typical in summer but the lowest values (2.4 m below soil surface) occurred in late autumn. During the seven experimental years, a general falling trend in GW was observed, especially in CONV. The farmers dredged the main drain in autumn 2014 and 2017, which was reflected as lower GW thereafter (Fig. 4). The highest GW was observed in CDI where the sub-irrigation helped to maintain the GW level. The GW was higher in CD than in CONV, but lower than in CDI, because damming by the regulation wells alone cannot raise GW in the field without replenishment of water by rainfall. Consequently, GW dropped to the critical Cg horizon for fewer days in CDI (137 days) and CD (259 days) than in CONV (631 days) during the whole study period (Nov. 2011–Dec. 2017). In 2015 and 2017, the main ditch dried up and CDI functioned like CD. However, in these years, the GW in CDI was higher than that in CD by an average of 18 cm. The highest annual mean difference between CDI and CONV was 66 cm (± 19 cm) and between CD and CONV 29 cm (± 16 cm) in 2012. The most acidic part of the Bg horizon (80–120 cm) and the entire BCg horizon (120–150 cm) were below GW and consequently saturated for 1315 days in CDI, 1135 days in CD, and 402 days in CONV. The median GW level was 92 cm, 100 cm, and 126 cm below the soil surface in CDI, CD, and CONV, respectively. All treatments differed significantly from each other (*p* < 0.001).

### Yields and offtake of nitrogen

**Fig. 5:**
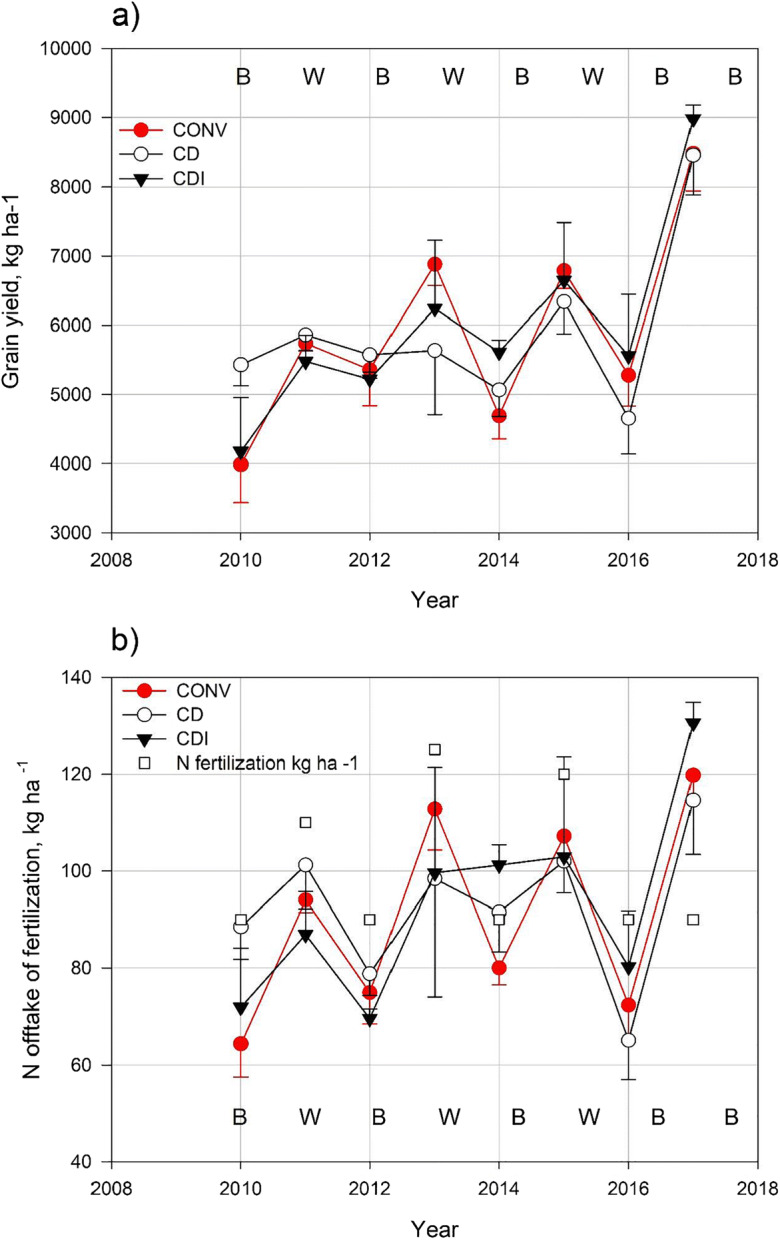
**a** Grain yield in the different water management treatments and **b** nitrogen (N) offtake in the harvested grain and amount of N applied with fertilizer. In 2010, conventional drainage (CONV) was applied also in the controlled drainage treatment (CD). In 2015 and 2017, the controlled drainage with sub-irrigation (CDI) treatment was identical to CD, because no sub-irrigation was applied. The error bars indicate standard deviation calculated from the results for the lower, middle, and upper section of each field

There were no marked differences in grain yield between the three water management practices, with the average yields (15% moisture) being 5900, 5880, and 5990 kg ha^−1^ in CONV, CD, and CDI, respectively, while the annual differences were much more pronounced (Fig. 5a). Straw yield, measured in 2010–2013, averaged at 2680–3100 kg ha^−1^ in different years and constituted 39% of aboveground dry matter. Offtake of N in harvested grain (Fig. 5b) averaged 92 kg ha^−1^ and corresponded to 92% of the amount applied in fertilization. Straw yield, analyzed for N only in 2010, contained 30.8 kg N ha^−1^ and represented 29% of total N uptake by the crop.

### Nitrogen in water samples

**Fig. 6:**
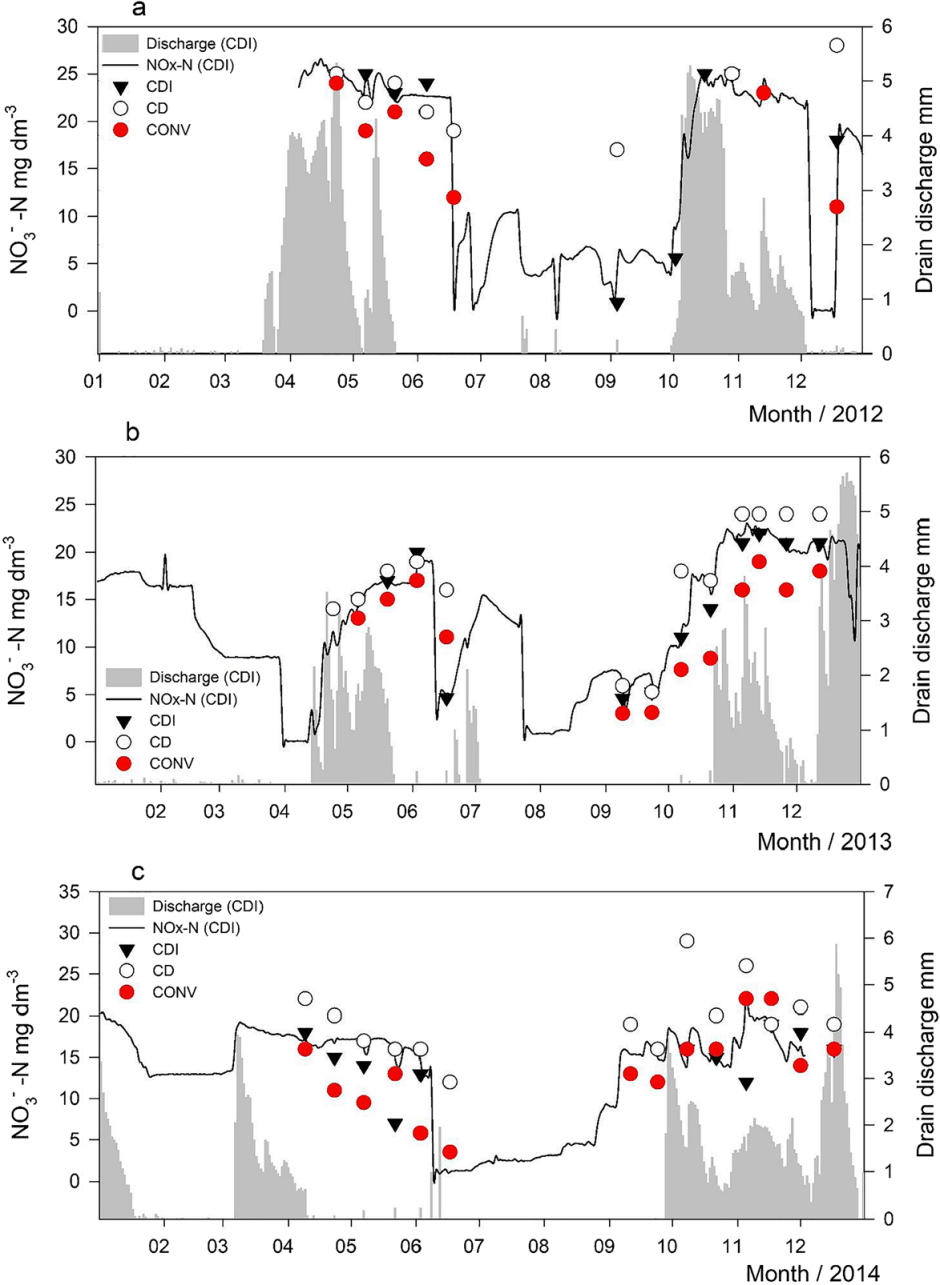
Concentration of nitrate-nitrogen (NO_3_^−^–N) in grab samples of discharge from the conventional subsurface drainage (CONV), controlled drainage (CD), and controlled drainage with sub-irrigation (CDI) treatments. The solid line shows the results of continuous measurements of NO_*x*_^−^–N (NO_3_^−^–N + NO_2_^−^–N) in a drainage well located in CDI. Gray represents discharge from CDI

In grab samples collected in spring and autumn 2011–2017, the median of NO_3_^−^–N concentration was 15.5 (range 0.32–31), 17.0 (2.0–29), and 13 (2.1–31) mg L^−1^ in CDI, CD, and CONV, respectively. The NO_*x*_^−^–N in drainage water was extremely high (up to 26 mg L^−1^) during high discharge in spring and autumn runoff, but decreased during the growing season (Fig. 6). In June, there was a sharp decrease in NO_*x*_^−^–N concentration immediately after pumping of ditch water with low NO_*x*_^−^–N concentration (< 5 mg L^−1^) into the control wells. During heavy floods caused by snow melt in spring, NO_*x*_^−^–N was also lower, since melt water and flood water diluted the concentration in the field. Continuously measured NO_*x*_^−^–N concentrations followed those of NO_3_^−^–N in grab samples (Fig. 6), the mean difference being 1 mg L^−1^ (SD 2 mg L^−1^).

**Fig. 7:**
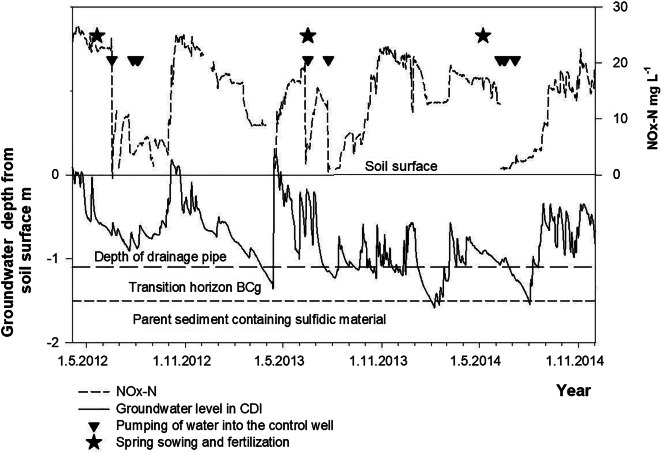
Time series of continuously monitored nitrous oxides (NO_*x*_^−^–N) and groundwater in the period April 2012–Nov 2014. Triangles denote time of pumping water in the controlled drainage treatment with sub-irrigation (CDI) and stars the time of fertilization

The concentration of NO_*x*_^−^–N increased when GW rose, with the exception of two seasons when a slight decrease took place (Fig. 7). The highest NO_*x*_^−^–N concentration (median 24 mg L^−1^) was found in autumns when the field flooded and GW was within 0–20 cm from the soil surface, but also when GW was at 80–120-cm depth (median 21 mg L^−1^). The EC of discharge water increased with decreasing GW, and obviously drain discharge increased when GW rose and ceased when GW dropped below the drainage depth.

**Fig. 8:**
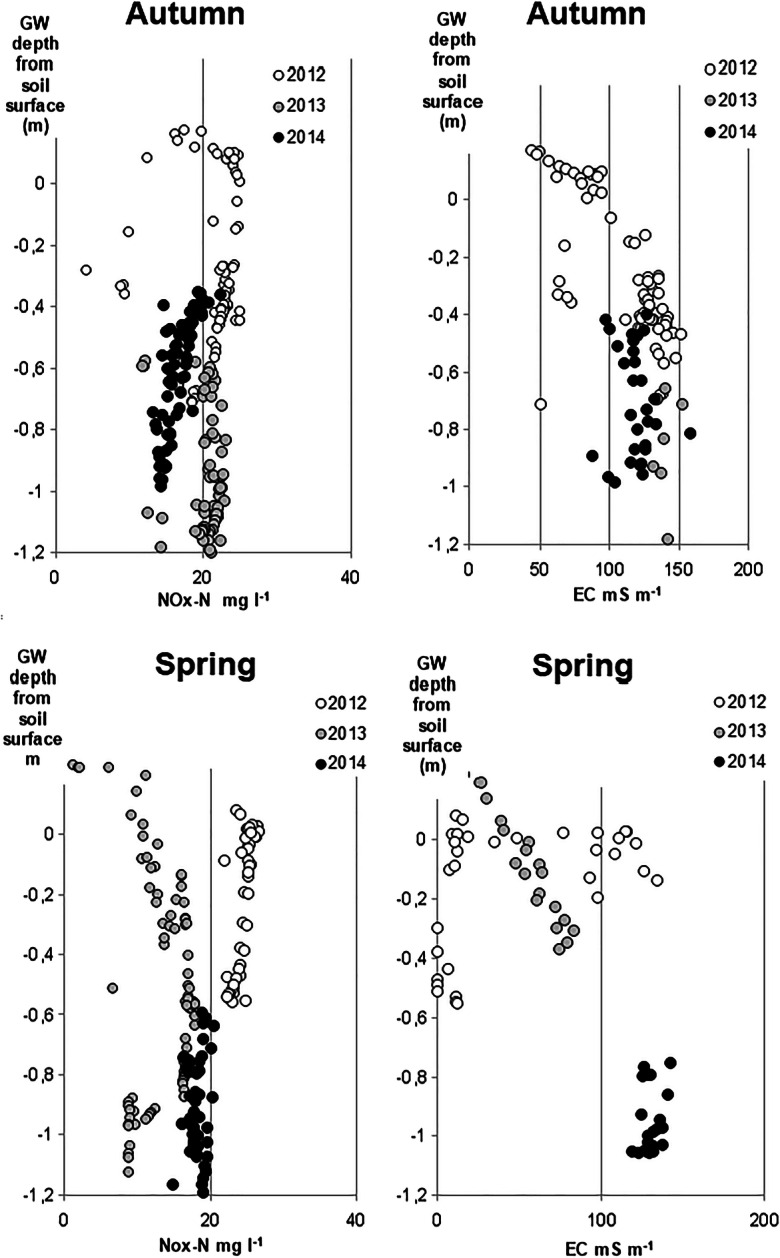
Concentration of (left) nitrous oxides (NO_*x*_^−^–N) and (right) electrical conductivity (EC) in discharge water, plotted against groundwater (GW) depth in spring and autumn 2012–2014. The measurements were carried out in the controlled drainage with sub-irrigation (CDI) treatment

The procedure for tracing the origin of leached NO_*x*_^−^–N was validated using data on continuously measured EC in discharge water and GW depth. EC was selected for the test because in active AS soils there is a very strong correlation between EC and SO_4_^2−^ in discharge water (e.g., Åström and Björklund 1997; Toivonen and Österholm 2011), an observation made also in our experimental area (Virtanen et al. 2016). In our study fields, oxidation of sulfidic material, producing SO_4_^2−^ and elevated EC in the discharge water, occurred mostly in the BCg horizon at 120–150 cm and in the upper part of the Cg (below 150 cm). Thus, the increasing EC in discharge with falling GW suggests that the discharge predominantly consists of water coming from the BCg and Cg horizons. Applying the same reasoning, the increasing NO_*x*_^−^–N upon falling GW and increasing EC (Fig. 8) suggests that NO_*x*_^−^–N also originates from the subsoil.

**Fig. 9:**
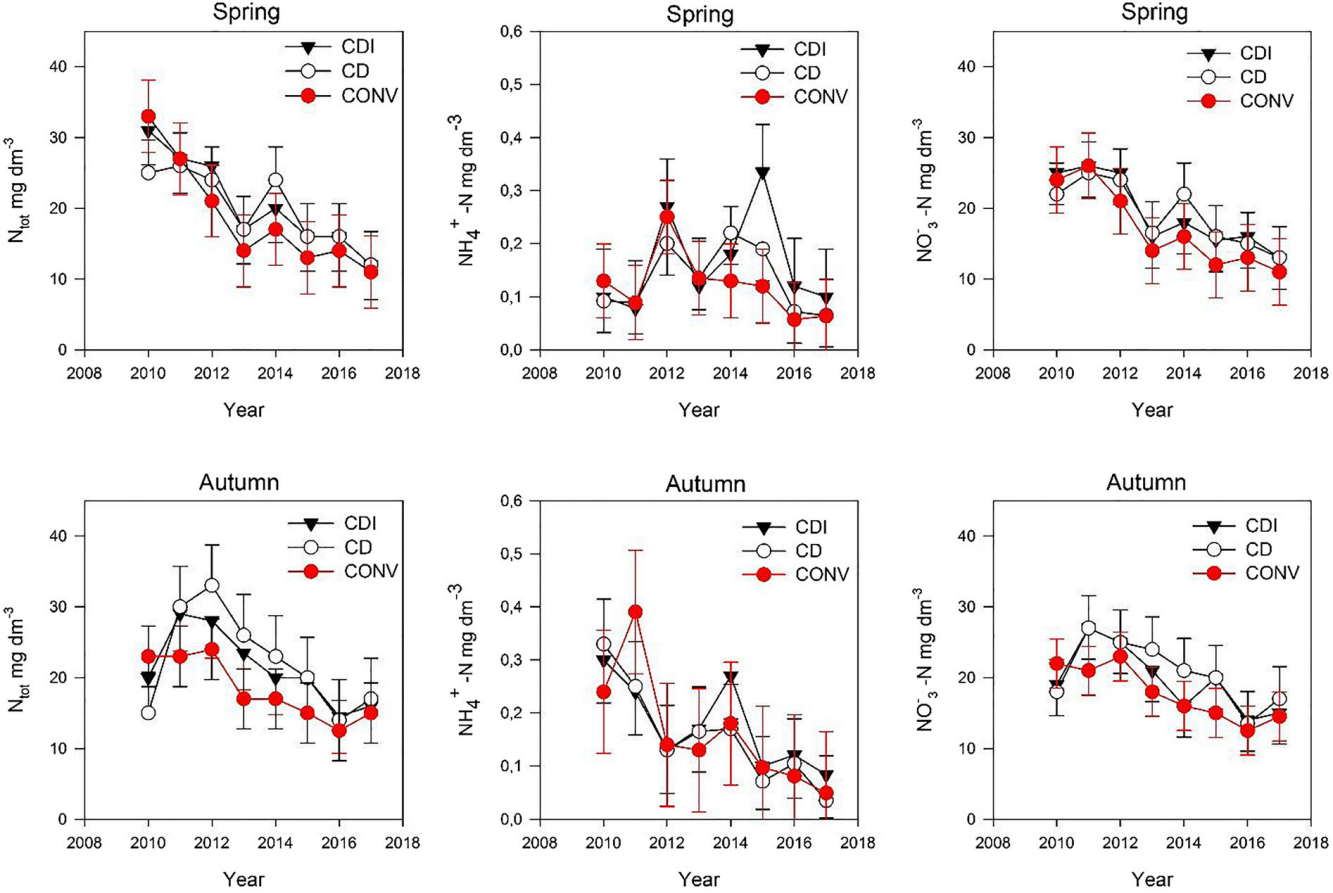
Concentrations of (left) total nitrogen (N), (center) ammonium-N (NH_4_^+^–N), and (right) nitrate-N (NO_3_^−^–) during (top) spring and (bottom) autumn runoff seasons, 2010–2017. The error bars indicate standard deviation of concentrations in each season

In spring discharge, Ntot and NO_3_^−^–N concentration in drainage water decreased steadily between 2010 and 2013 and more irregularly and more slowly thereafter (Fig. 9). In autumn 2010, there was an increase, and after 2012 a decrease, in Ntot concentration in drainage water. On average, 91% of Ntot consisted of inorganic N forms (NO_3_^−^–N, NO_2_^−^–N, and NH_4_^+^–N). Nitrate was the dominant N form, with NH_4_^+^–N comprising less than 1% of Ntot and with NO_2_^−^–N concentrations being negligible. There was a tendency in autumn throughout the experimental period for Ntot concentrations to be higher in CD and CDI than CONV. In spring, the difference between the fields was less pronounced. The differences in Ntot were attributable to lower NO_3_^−^–N concentrations in water coming from CONV.

### Nitrogen load

The annual variation in N loads within a given experimental year was much higher than that between the three fields. The annual Ntot load was 50 kg ha^−1^ (range 35–71 kg ha^−1^) in CONV, 52 kg ha^−1^ (range 31–81 kg ha^−1^) in CDI, and 63 kg ha^−1^ (range 36–91 kg ha^−1^) in CD (Table 3). Thus, the loads calculated for CD were around 26% higher compared to CONV (*p* = 0.0041) and CDI (*p* = 0.0053). This difference was consistent and can be connected to the amount of discharge which was about 15% higher in CD than in CONV and CDI. The difference was apparent also in summer 2015 and 2017, when sub-irrigation could not be performed and the CDI field was thus managed like CD. Higher discharge (and Ntot load) can therefore be regarded as a characteristic of the particular field. There was a positive correlation (*r* = 0.65, *p* = 0.0013; excluding 2015 *r* = 0.81, *p* < 0.001) between Ntot load and discharge, and a negative but non-significant correlation (*r* = − 0.39, *p* = 0.084) between Ntot load and N offtake in the harvested grain. The year 2016 was an outlier, with both Ntot load and N offtake in the harvested grain being small. In that year, a wet summer contributed to low yields and N offtake while a very dry autumn was conducive to low Ntot load. Excluding that year, the correlation between these variables increased to *r* = − 0.80 (*p* < 0.001).

**Table 3:** Annual discharge and loads of total nitrogen (N) and inorganic N species. The column NO_*x*_^−^–N shows load estimates based mainly on the results of continuous measurements in the controlled drainage with sub-irrigation (CDI) field. In 2015 and 2017, additional water was not pumped into CDI pipes, and CDI was treated similarly to CD

Year	Discharge, mm			Total N, kg ha^−1^			NO_3_^−^–N, kg ha^−1^			NH_4_^+^–N, kg ha^−1^			NO_2_^−^–N kg ha^−1^			NO_*x*_–N kg ha^−1^
	CDI	CD	CONV	CDI	CD	CONV	CDI	CD	CONV	CDI	CD	CONV	CDI	CD	CONV	
2011	257	292	244	67	81	68	61	73	62	0.30	0.42	0.38	0.084	0.063	0.080	-
2012	310	336	278	81	91	71	74	84	65	0.63	0.69	0.43	0.074	0.045	0.014	64^a^ + (5)
2013	236	281	260	49	60	44	45	56	43	0.36	0.43	0.32	0.037	0.044	0.010	41^b^
2014	257	333	278	48	74	52	41	70	48	0.66	0.59	0.38	0.029	0.039	0.014	34^c^ + (10)
2015	298	348	322	53	60	48	51	57	45	0.91	0.69	0.42	0.015	0.012	0.007	–
2016	244	265	250	34	41	35	33	39	33	0.28	0.22	0.15	0.031	0.060	0.043	–
2017	204	233	248	31	36	35	30	35	35	0.18	0.14	0.11	0.009	0.009	0.009	–
Mean	258	298	268	52	63	50	48	59	47	0.47	0.45	0.31	0.040	0.039	0.025	

^a^Continuous measurements between April 3 and December 31, 2012, and (in brackets) grab sampling between January 1 and April 2, 2012

^b^Continuous measurements throughout the year 2013

^c^Continuous measurements between January 1 and December 3, 2014, and (in brackets) grab sampling between December 4 and 31, 2014

There was a good match between the load estimates calculated on the basis of manual sampling and predominantly continuous sampling (Table 3). In 2013, in particular, when continuous measurement ran throughout the year, the estimates obtained from continuous sampling were 9% smaller than those derived from manual sampling. On this basis, it can be concluded that the annual Ntot load estimates based on manual sampling were within ±10% of the correct value.

### Nitrous oxide emissions

**Fig. 10:**
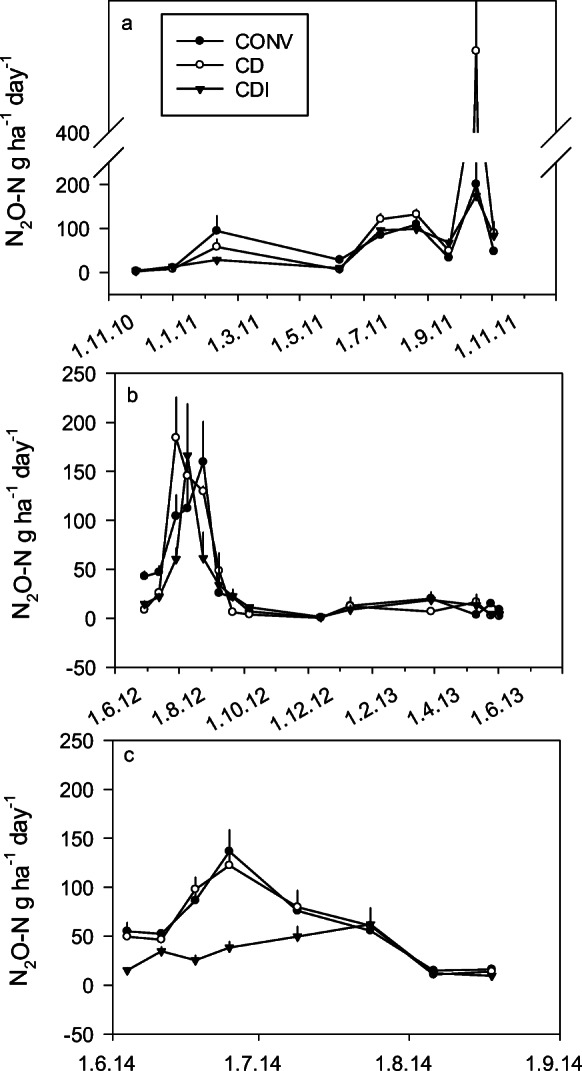
Emissions of nitrous oxide (N_2_O) from the experimental plots in **a** 2010–2011, **b** 2012–2013, and **c** 2014. Fertilization events were 10.5.2011, 21.5.2012, 16–17.5.2013, and 8.5.2014

Mean daily emission rate of N_2_O in 2010–2014 was 55, 64, and 42 g N day^−1^ in CONV, CD, and CDI, respectively. The fluxes of N_2_O peaked in early summer, but also at times of frost initiation, as in January 2011, and on sudden increases in soil moisture due to high GW, as in autumn 2011 (Fig. 10). The annual rate ranged from 7.9 to 28 kg N ha^−1^ (Table 4). Mixed model analysis suggested that over all years, CD and CDI lowered the N_2_O flux compared with the CONV treatment (*p* = 0.0003) although the difference was evident only for CDI in summer 2014 (Fig. 10).

**Table 4:** Nitrogen balance (kg N ha^−1^ year^−1^) for the fields in the two years in which an estimate of annual N_2_O emissions was available. CONV = conventional subsurface drainage, CD = controlled drainage, CDI = controlled drainage with sub-irrigation

	2011, wheat			2012, barley		
	CONV	CD	CDI	CONV	CD	CDI
Fertilization	+ 110	+ 110	+ 110	+ 90	+ 90	+ 90
N in grain	− 87	− 101	− 94	− 70	− 79	− 75
Leaching	− 68	− 81	− 67	− 71	− 91	− 81
N_2_O	− 24	− 28	− 20	− 10	− 9	− 8
N balance	− 69	− 100	− 70	− 61	− 89	− 74

**Fig. 11:**
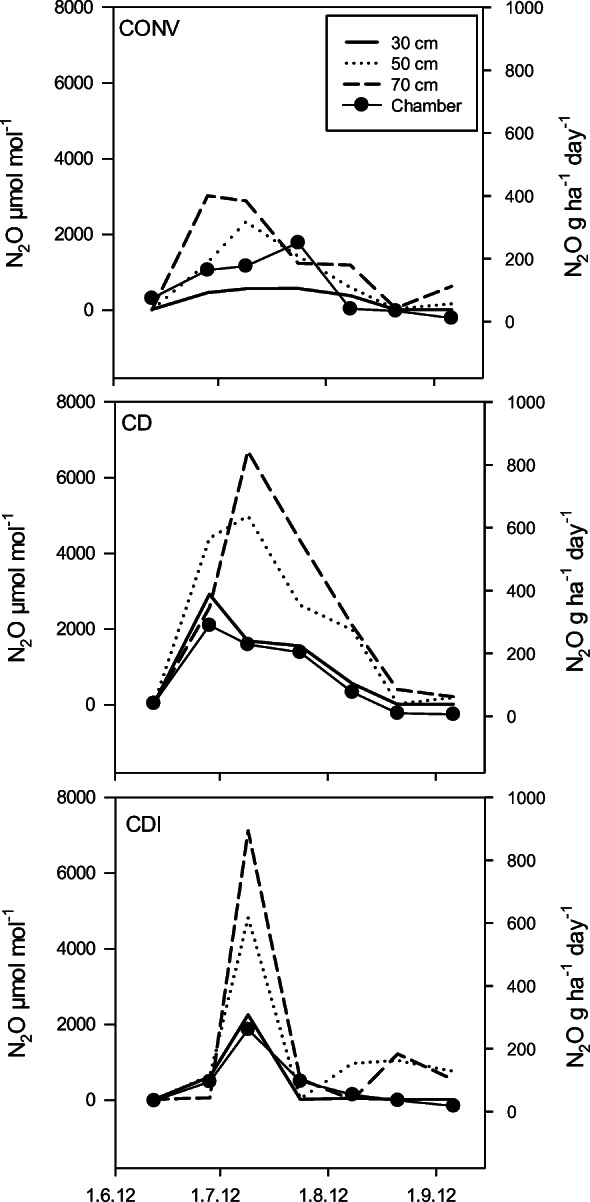
Nitrous oxide (N_2_O) flux measured from chamber and the concurrent concentration of N_2_O in soil air at 30-cm, 50-cm, and 70-cm depth in summer 2012 in the conventional subsurface drainage (CONV), controlled drainage (CD), and controlled drainage with sub-irrigation (CDI) treatments

The concentration of N_2_O in the soil profile increased with increasing depth from 30 to 70 cm (Fig. 11). The CONV field generally showed the lowest concentration, and the CD field the highest, and a seasonal peak in N_2_O was observed in July. The flux measured from the adjacent chambers followed the pattern of the concentration changes in the 30-cm layer.

**Fig. 12:**
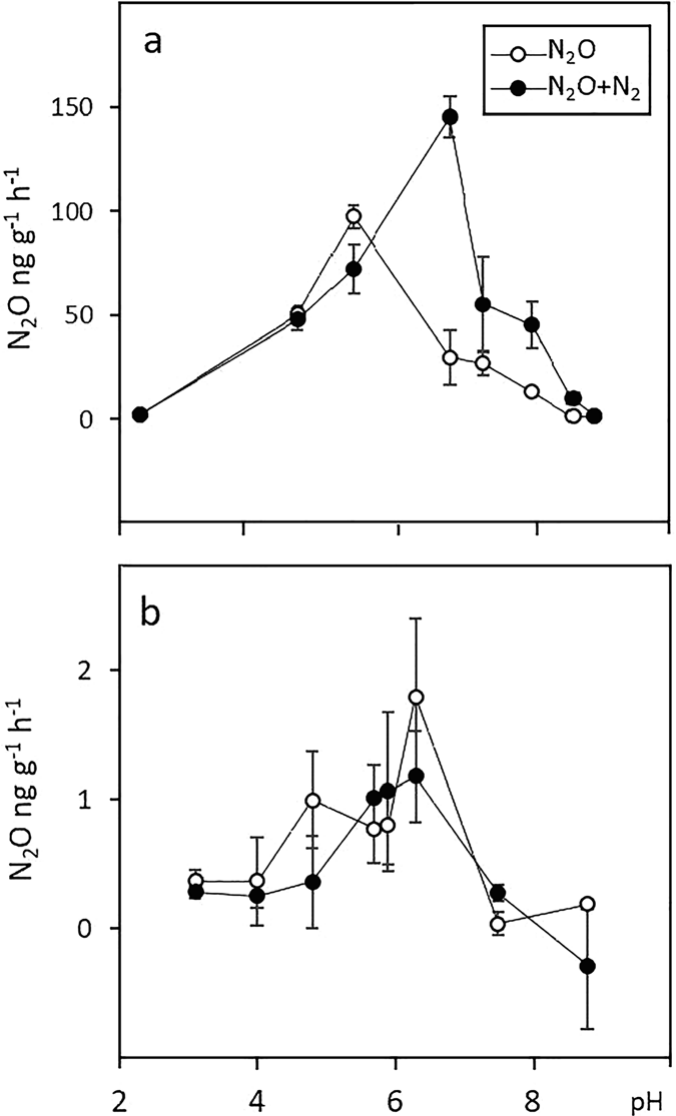
Effect of pH on denitrifying enzyme activity (DEA) in layers 0–20 cm (**a**) and 80–100 cm (**b**)

In the 0–20-cm soil layer of CONV, maximum N_2_O production was observed at pH 5.3 and maximum N_2_O + N_2_ at pH 6.8 (Fig. 12). Below pH 5, there was no N_2_ production whereas at pH 6.8 most of the N production was emitted as N_2_. In the deeper soil layer, the maximum DEA was measured at pH values around 6 and the proportion of N_2_ in the total DEA was negligible at each pH level. The results with and without acetylene differed significantly in the topsoil (*p* < 0.05) but not in the deeper layer.

### Nitrogen balance

A comprehensive N balance was estimated for the 2 years in which N_2_O emissions were measured (Table 4). Because straw was returned to the soil, straw N content (about 30 kg ha^−1^) was not taken into account in the balance calculation. The grain contained 9–23 kg ha^−1^ less N than was applied with fertilizer. Due to the abundant losses of N through leaching and emissions, the Ntot removal was clearly higher than the N addition rate. In both years, net removal of N was highest in the CD treatment where the N discharge was higher than that in the other two treatments.

During the entire period 2011–2017, leaching and N offtake in the harvested grain, both measured every year, averaged 150 kg ha^−1^ (range 106–182 kg ha^−1^), which represented 148% of N fertilization, i.e., 48 kg ha^−1^ (range 16–80 kg ha^−1^) more than what was applied in N fertilizers (see Table 3 and Fig. 5). Assuming the average N_2_O emissions of 16 kg ha^−1^ also for 2012–2017 and including these in the calculation, net removal of N from the field was on average 64 kg ha^−1^ (range 32–96 kg ha^−1^), with the result being consistent from year to year. This shows that the study soil has a very high N supply capacity.

## Discussion

Nitrogen stock in the soil, along with N fertilizers, is the source of mobile N. At 0–100 cm in soil at our study site, the Ntot stock (19.5 t ha^−1^) was equal to or larger than that reported for eight mineral soils of Denmark, Finland, Norway, and Sweden (range 7.5–19.5 t ha^−1^, mean 14.0 t ha^−1^) (Lindén et al. 1992a). As it is closely associated with soil organic matter, the Ntot stock usually decreases with depth in non-AS mineral soils (Lindén et al. 1992a). This was not the case in the AS fields studied here, where the Ntot stock at 100–200 cm was 36% larger than that in the uppermost 100 cm. In this respect, the Ntot stock in our AS soil resembles the value reported for other Finnish AS soils and differs from non-AS soils (Paasonen-Kivekäs and Yli-Halla 2005; Šimek et al. 2011).

The large Ntot stock and low C/N ratio suggest substantial potential for N mineralization throughout the soil profile, but waterlogging likely prevents N mineralization below the drainage pipes for most of the year. However, the GW level fell to 2–2.5 m periodically in summer, allowing aerobic reactions to take place. Together with near-neutral pH in those horizons that have not yet been acidified by the oxidation of sulfidic materials, the oxic conditions may enable periodic activity of the microbial community conducive to N mineralization.

Our experimental field seems not to contain an exceptionally large Nmin stock within the rooting zone. The Nmin stock (42.2–43.2 kg ha^−1^) within the top 40 cm in 2010 and 2011 was at the upper end of the range found at 0–40 cm in eight mineral soils in Denmark, Finland, Norway, and Sweden (17–48 kg ha^−1^, mean 31 kg ha^−1^) (Lindén et al. 1992b) and in the lower part of the range in 2012 and 2013. The annual differences in Nmin stock within the topsoil (0–40 cm) were attributable to NO_3_^−^–N, while NH_4_^+^–N levels were more constant. In contrast, the Nmin stock increased with depth in the soil horizons that are mostly in the reduced state, with NH_4_^+^–N as the sole Nmin species in those horizons, agreeing with results from other AS soils of Finland (Paasonen-Kivekäs and Yli-Halla 2005; Šimek et al. 2011). The large Nmin stock in AS subsoil and parent sediment, besides the presence of sulfidic material, was confirmed as the most prominent difference between AS soils and non-AS soils.

High N concentration in drainage water during the first few years, twice as high as during the last years of monitoring, coincided with high Nmin in the top 40 cm of the soil. As found in the top 40 cm, NO_3_^−^–N was the dominant N form in drainage water. High initial concentrations may have been caused by oxidation-induced N mineralization associated with soil disturbance during construction of the experimental field in 2010. Peaks in N concentration in drainage water can also occur in practice in the year following ditching activities (Äijö et al. 2016). Thus, starting from 2012, the N concentration in drainage water can be taken to represent the long-run loading from a non-disturbed AS soil. The NO_3_^−^–N concentration in discharge was at the same level as in 5.5-year monitoring of an AS soil at Ilmajoki about 60 km away from our study site (Bärlund et al. 2004). In that study, NO_3_^−^–N concentration was mostly around 12 mg L^−1^. In a 3-year monitoring (1991–1993) of spring cereal plots in a non-AS clay soil at Jokioinen, Finland, where the SOC content in the subsoil between 35 and 210 cm was 0.3–0.5%, the average Ntot concentration in drainage water was found to be only 6.1 mg L^−1^, and 85% of Ntot was in the form of NO_3_^−^–N (Turtola and Paajanen 1995). Likewise, in an agricultural mineral soil catchment in SW Norway, the average NO_3_^−^–N concentration in 1994–2016 was 4.3 mg L^−1^, comprising 78% of Ntot concentration (Chen and Bechmann 2019). The soil at our study site thus produced drainage water with substantially higher N concentrations than non-AS soils.

In this study, Ntot load through drainage pipes averaged 48 kg ha^−1^ in 2012–2017, while annual load from the three individual fields were 31–91 kg ha^−1^. Even the lowest annual N loads were at least double those from a clay soil cropped with spring cereals in a 7-year study in Jokioinen, Finland (Jaakkola 1984), or estimated as a specific N load from agricultural land in Finland (15 kg ha^−1^; Tattari et al. 2017). Owing to the different water management systems in the fields, the Cg horizon was exposed to oxic conditions for a longer time in CONV than in CD and CDI. However, although the time available for mineralization of organic matter was shorter in CD and CDI, N leaching was at the same level in CDI as in CONV, while the more abundant leaching from CD can be explained by the higher amount of discharge. This outcome was surprising, because in other studies on CD or CDI in non-AS soils that probably had low Nmin stocks in the subsoil, N leaching declined significantly under these water management practices due to decreased runoff (e.g., Evans et al. 1995; Woli et al. 2010; Carstensen et al. 2019). Moreover, it is likely that the large Nmin stock already present in the soil influenced N leaching more than production of new Nmin by mineralization. Our results thus confirmed hypothesis 1, of abundant N leaching from this AS soil, but did not support hypothesis 3, of decreased N leaching with elevated GW level.

We were unable to clearly identify the soil horizon(s) acting as the main sources of the large N load entering drainage waters. The high concentration of Nmin in the top 40 cm of soil likely contributed to the abundant N load during the first few years explaining at least partly the declining trend in N loads during the experimental period. An influence of subsoil Nmin reserves is suggested by the finding that, whenever GW dropped into the subsoil, discharge thereafter contained a high NO_*x*_^−^–N load. The explanation may lie in the large stock of NH_4_^+^–N in the anoxic subsoil. If discharge water contains N originating from this NH_4_^+^–N stock, nitrification must have occurred in situ in the near-neutral Cg horizon during the oxic periods in summer or at some stage of the transport process. When saturated conditions return, the NO_3_^−^–N formed and dissolved in pore water can be transported into the drainage pipes above and out of the soil, explaining the high N load to drainage waters.

Mean flux of N_2_O (54 g N ha^−1^ day^−1^) was high compared with that in Finnish mineral soils in general, where the mean emission rate with annual crops is around 10 g N ha^−1^ day^−1^ (Regina et al. 2013). It was also high compared with organic soils, where a typical rate in annual crop cultivation is 30 g N ha^−1^ day^−1^ (Maljanen et al. 2007). Extremely high N_2_O emissions (up to 60 kg N_2_O–N ha^−1^) from AS soils have also been found in Australia (Denmead et al. 2010) and Denmark (Petersen et al. 2012). The annual flux rates at our site (8–28 kg N_2_O–N ha^−1^) were based on only 9–15 measurements per year and can be considered indicative rather than absolute. However, the high values found in all three growing seasons indicate that the abundant N stock in these soils is a major source of N to watercourses and to the atmosphere, supporting our hypothesis 2.

Hypothesis 4, that keeping the sulfidic layer inundated decreases N_2_O emissions, was supported by the data, but the effect was not consistent over the years. Significant production of N_2_O usually requires conditions allowing both nitrification and denitrification to occur simultaneously, with the final emission rate depending on the proportions of N_2_O and N_2_ in the end products of denitrification (Bollmann and Conrad 1998; Davidson 1991). Inundation can reduce N_2_O emissions via two potential mechanisms: (i) by retarding nitrification in the layer with high NH_4_^+^–N content and (ii) by extending the layer supporting N_2_O reduction to N_2_. The finding that the highest N_2_O concentration in soil air was measured at the lowest depth monitored (70 cm) suggests that the source of NO_3_^−^–N was in the deeper soil layers where nitrification may occur during drought periods and the NO_3_^−^–N formed can be available for further denitrification as the GW level rises. The active production zone of N_2_O was typically close to the GW depth, in the zone of varying moisture conditions, as also found in a study on a buffer wetland (Saari et al. 2013).

High emissions of N_2_O have also been linked to more abundant microbial populations in AS soils than non-AS mineral soils, at least in the subsoil (Šimek et al. 2011, 2014). In our soil, however, the basal respiration rates were lower (data not shown) than reported by Šimek et al. (2014). The denitrifying enzyme activity (DEA) was also lower than that in the AS soil studied by Čuhel and Šimek (2011). Production of N_2_O could also be purely chemical, in which case N_2_O would be formed from hydroxylamine or nitrite especially in acid soils (Zhu-Barker et al. 2015). This was suggested as a likely mechanism in a Danish AS soil (Taghizadeh-Toosi et al. 2019). However, in our soil, nitrite was likely present only in negligible amounts as its concentration in drainage water was low. The concentration measurements on soil air suggested that there was high N_2_O production in soil below 30 cm, and thus the low pH (3.8–4.8) in the Bg and BCg horizons may be partly responsible for the high N_2_O emissions. Low pH inhibits N_2_O reductase, which increases the proportion of N_2_O in the end products of denitrification (Thomsen et al. 1994). This appears to be a plausible explanation for the high N_2_O emissions in our study, and the DEA results showed lack of N_2_ production at the lowest pH levels. It is also possible that the microbial populations in the subsoil lack N_2_O reductase in general, as the DEA results showed a total absence of N_2_ within the end products of denitrification in the subsoil. Only around 65% of denitrifying organisms carry N_2_O reductase (Philippot et al. 2011) and another possible explanation for the high N_2_O emissions could be that such bacteria dominate in the study soil, but this could not be verified from the results.

Grain yields on the study soil (mean 6200 kg ha^−1^ for wheat and 5800 kg ha^−1^ for barley) far exceeded the national average yields of these crops (3700 and 3500 kg ha^−1^, respectively) and resulted in high N uptake. The harvested grain and straw contained more N than was applied in the fertilizer, e.g., the N content of grains alone was on average 92% (annual range 81–135%) of the N dose supplied by fertilizer. This exceeds the 75–78% commonly reported for spring cereals in Finland at similar fertilization levels (Esala and Larpes 1986; Rajala et al. 2007). However, in our experimental setup, there were no plots without N fertilization, preventing us from drawing firm conclusions on the role of ample native N supply in high yields and N offtake, which may also be attributable to good soil structure and favorable water management.

When N losses to water and to the atmosphere were taken into account, the difference in N output between our AS soil and non-AS soils was even more drastic. In our experiment, N leaching plus N offtake in the harvested grain exceeded N fertilization in every year studied, the average excess being 48 kg ha^−1^ while in an 11-year field experiment on a non-AS clay soil in Jokioinen, Finland, the harvested grain and drainage waters contained 27 kg ha^−1^ less N than was applied in fertilizer (Salo and Turtola 2006). There was thus a 75 kg ha^−1^ difference in N output between the two experiments. The difference increases to 89 kg ha^−1^ when considering the average N_2_O–N emissions of 16 kg ha^−1^ in our experiment and 3.5 kg ha^−1^ in conventional cereal cultivation (Syväsalo et al. 2006). These results demonstrate that AS soils contain an exceptionally large N stock which supplies N to the crop and is a source of N emissions and leaching.

## Conclusions

Large Ntot and Nmin stocks, particularly in subsoil subjected to alternating redox conditions, are a specific characteristic of AS soils compared with non-AS mineral soils. In the AS soil in this study, abundant transport of N was shown to occur through different ways, including offtake in the harvested crop, leaching, and gaseous losses, all exceeding those in non-AS mineral soils and resulting in negative N balance. There was a large NH_4_^+^–N stock in the subsoil, and it may contribute to N fluxes through nitrification during short oxic periods, with subsequent transport of NO_3_^−^–N or denitrification to N_2_O or N_2_. The N_2_O emissions from the study soil were extremely high, which is potentially explained by lack of N_2_O reduction in deeper soil layers due to low pH or unfavorable composition of the microbial population. The study provided only moderate evidence that manipulation of groundwater level by controlled drainage or sub-irrigation can reduce N mineralization and N_2_O emissions and thus alleviate some environmental consequences of cultivation of AS soil. Having identified the large N flows and stocks, we now need to monitor the Nmin stock spatially and temporally in more detail. Relationships between the large NH_4_^+^–N stock, NO_3_^−^–N leaching, N_2_O emissions, and N offtake by the crop should be experimentally investigated and presence of N_2_O reductase in the microbial population needs to be examined. Whether N fertilization rates to AS soils can be lowered is also an urgent and practical topic for further research. Our results strongly suggest that the large N stock in AS soils should be taken into account when planning fertilization.
